# Star of the Diabetic Macular Show: Stellate Nonhereditary Idiopathic Foveomacular Retinoschisis (SNIFR)

**DOI:** 10.18502/jovr.v20.15125

**Published:** 2025-05-05

**Authors:** Cory A. Christensen, Neha Gupta, Mark P. Breazzano

**Affiliations:** ^1^Department of Ophthalmology & Visual Sciences, SUNY Upstate Medical University, Syracuse, NY, USA; ^2^Department of Ophthalmology, Icahn School of Medicine at Mount Sinai and New York Eye and Ear Infirmary, New York, NY, USA; ^3^Flaum Eye Institute, Department of Ophthalmology, University of Rochester School of Medicine & Dentistry, University of Rochester Medical Center, Rochester, NY, USA

##  PRESENTATION

**Figure 1:**
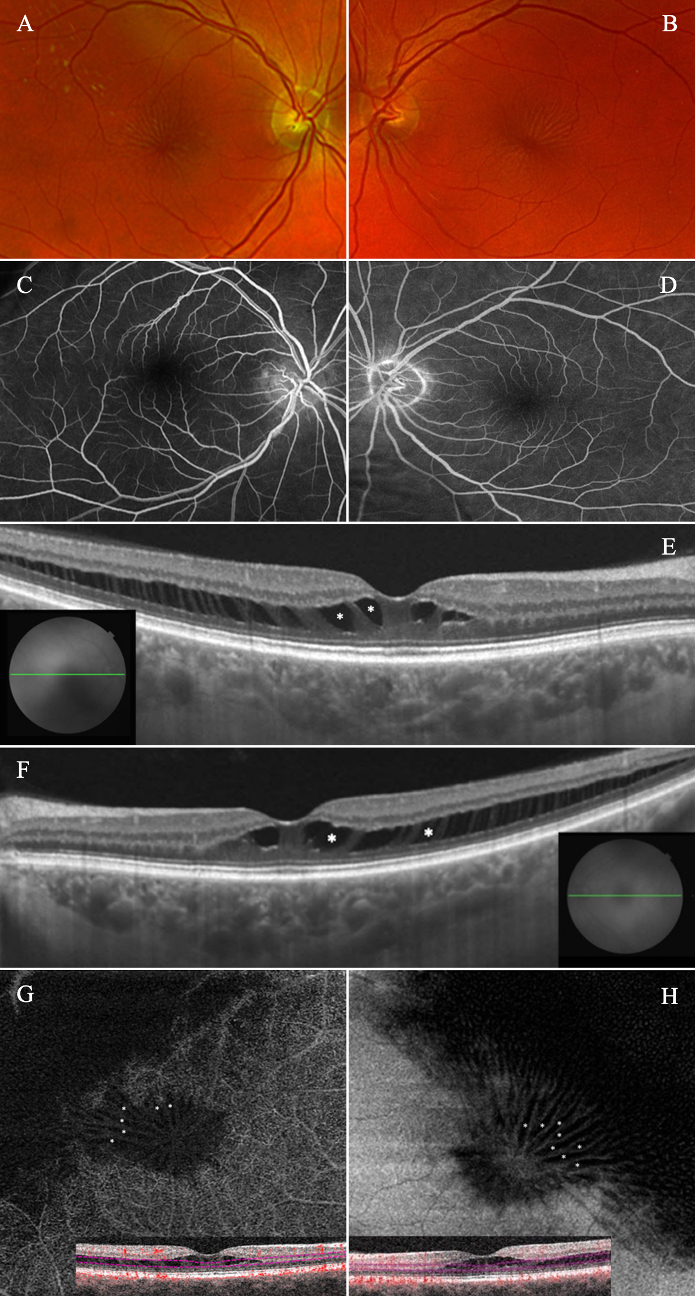
(A & B) Fundus photography of the right and left eyes, respectively, with evidence of retinal folds radiating from the foveae. (C & D) Intravenous fluorescein angiography in late phases of the right and left eyes, respectively, with no evidence of staining, leakage, or microaneurysms, despite the history of diabetes mellitus. (E & F) Swept-source optical coherence tomography B-scan through the foveae of the right and left eyes, respectively, revealing hyporeflective cavities (asterisks) involving the outer plexiform layers with extension into the temporal maculae. (G & H) Spectral-domain optical coherence tomography angiography of the right and left eyes, respectively, displaying an absence of flow signal through pseudocystic spaces in stellate configurations (asterisks) upon en-face structural imaging with segmentation through the outer plexiform layers (pink lines, B-scans).

A 66-year-old man with recently diagnosed type 2 diabetes mellitus presented with concern for macular edema. He reported no visual complaints and had no family history of ocular disease. Examination revealed bilateral 20/20 Snellen acuity, with blunting of the foveal reflexes and macular star-like appearances [Figures 1A & 1B]. Intravenous fluorescein angiography in late phase revealed no evidence of staining or leakage [Figures 1C & 1D]. Swept-source optical coherence tomography (OCT) showed perifoveal hyporeflective cavities OU [Figures 1E & 1F]. Spectral-domain OCT angiography (OCTA) revealed classic stellate patterns of radially orientated macular spoking and lack of flow signal within the pseudocystic spaces, indicative of avascular outer plexiform layer [Figures 1G & 1H].

##  DISCUSSION

Patients with a history of diabetes and OCT findings of perifoveal pseudocystic spaces can easily bias the clinician toward diabetic macular edema. In this case, a negative family history, lack of diabetic retinopathy, presence of macular pseudo-folds, and characteristic OCT and structural OCTA findings were consistent with stellate nonhereditary idiopathic foveomacular retinoschisis (SNIFR).

OCT alone helps exclude vitreomacular interface abnormalities for tractional causes of foveoschisis.^[1]^ However, OCTA adds utility in distinguishing SNIFR from X-linked retinoschisis, which classically demonstrates vascularity from the flow signal within the outer plexiform layer.^[2]^ Taxane- and niacin-induced retinoschisis should be excluded with history-taking, and as in this case, similarly lack leakage on intravenous fluorescein angiography.

##  Ethical Considerations

Institutional Review Board approval and associated contingencies were waived per guidelines for this case report as it does not qualify as human subjects research.

##  Financial Support and Sponsorship

None.

##  Conflicts of Interest

MPB has served as an advisor for Iveric Bio/Astellas and as a consultant for ONL Therapeutics.
